# 27363 Forging Collaboration and the Scalable Dissemination of Biomedical Research Commercialization Education

**DOI:** 10.1017/cts.2021.553

**Published:** 2021-03-30

**Authors:** Samantha Cook, Stefan Koehler, Janani Ramaswamy, Kristen Wolff, Michelle Larkin, Jeanne Wright, Mona Bruch Moore, Jon Servoss

**Affiliations:** 1 Michigan Medicine, University of Michigan; 2 University of Michigan

## Abstract

ABSTRACT IMPACT: A robust and collaborative network of expertise and services is essential for successful research commercialization, including timely and scalable educational support for CTSA institutions and individual faculty investigators with biomedical innovations. OBJECTIVES/GOALS: Leverage expertise at the University of Michigan (UM) by creating collaborative and scalable interactive online courses to instruct and prepare internal and external faculty to navigate critical stages of life science academic research commercialization. METHODS/STUDY POPULATION: UM’s Fast Forward Medical Innovation created two online courses with the UM Office of Technology Transfer and the Michigan Institute for Clinical & Health Research (MICHR). Collaborative planning committees, with content and educational experts, set course goals and learning objectives based on audience needs (e.g. preparation for consultations, commercialization concepts, etc.). Draft content was developed, peer reviewed, and revised before Articulate Storyline was used to convert didactic content to active learning content (e.g. interactive slides, scenarios, quizzes, and forms). Pilot testing was conducted prior to the launch to faculty investigators throughout the UM network. RESULTS/ANTICIPATED RESULTS: Intellectual Property in the Academic Setting launched via the FFMI website and newsletter in July 2020 and has had 66 learners to date. Medical Device Regulations launched in October 2020 and has 22 learners. OTT and MICHR have successfully integrated the courses into their consultation process by requesting review from faculty investigators. We suspect that this will lead to more in-depth and meaningful conversation. Additionally, these courses have been integrated into an FFMI commercialization course to instruct on critical concepts. Evaluation and refinement for both use cases will ensue, as well as inform future collaborative courses. DISCUSSION/SIGNIFICANCE OF FINDINGS: Early results suggest that the courses are advantageous and can serve as a model for future collaborations. The opportunity to disseminate the courses across the CTSA network, as well as collaborate with other institutions, to scale localized expertise to a broader network is promising.

